# When Utilitarian Claims Backfire: Advertising Content and the Uptake of Insects as Food

**DOI:** 10.3389/fnut.2018.00088

**Published:** 2018-10-02

**Authors:** Sebastian Berger, Christian Bärtsch, Christina Schmidt, Fabian Christandl, Annika M. Wyss

**Affiliations:** ^1^Department of Organization and Human Resource Management, Institute of Organization, University of Bern, Bern, Switzerland; ^2^Essento Food AG, Zurich, Switzerland; ^3^University of Cologne, Cologne, Germany; ^4^School of Psychology, Fresenius University of Applied Science, Cologne, Germany

**Keywords:** entomophagy, marketing, consumer behavior, sustainable food, disgust

## Abstract

A key challenge for climate change mitigation on the consumer side is to break habits that excessively lead to carbon emission. One of the culturally most robust human routines is the heavy reliance of the Western societies on conventional meat sources such as beef, pork, and poultry, which were recently accused of causing particularly high climate costs. In this light, the UN (FAO) has suggested the increasing use of insects as an alternative source of animal protein intended for human diets. Yet, insects have not reached the mainstream of Western cuisine. Currently, a frequent promotion strategy of insects is to highlight the Utilitarian benefits associated with their consumption (e.g., with respect to the environment or one's health). The present research addresses the efficacy of such claims in a consumer research study involving 180 participants recruited from the general population in Germany. Arguing based on social-cognitive research in the area of moral and environmental psychology, we hypothesized and found that a focus on beneficial, but temporally distant motives (e.g., health)—counterintuitively—decreases consumption in comparison to immediate, hedonic advertisements (e.g., tasty). Furthermore, our study provides process evidence suggesting pretrial expectations induced by a particular claim mediate the relationship between claims and consumption. Thus, the present research not only refutes a state-of-the-art approach in the promotion of insects as food, but also provides an alternative approach and process evidence by integrating psychological factors.

## Introduction

The increasing concern about the impact of our modern lifestyles on the earth's ecosystem has led to immense efforts to address climate change. Globally, food production accounts for about a quarter of all anthropogenic greenhouse gas emissions (1), and the upward trend is expected to continue. Research on climate cost of conventional foods emphasizes the problematically high level of conventional meat intake (2). Insect-based consumption has been suggested as a more sustainable (e.g., less carbon dioxide emissions and lower water footprint) and healthy (high in protein, fats, minerals, and vitamins) way of consuming animal protein (3–5), with high economic value (6). In their report, the United Nations (7), systematically compare nutrients and climate costs of various insect species against conventional meat sources and conclude that insects are indeed a viable alternative source of animal protein.

The rising interest in entomophagy (i.e., insect consumption by humans) results not only from the increased attention paid to anthropogenic climate change but also from recent advances in agricultural technology and food safety, which make insects a viable option for industrial and private production. This enables innovators from top-level cuisine just like several young companies to enter the market for insects as food, offering various consumer products ranging from luxurious options to mass-market alternatives. However, despite these advances and the environmental benefits, insects are rarely eaten in Western countries and the confrontation with insect-based food often evokes skepticism and disgust (8). Disgust is primarily a result of social and cultural learning but also has trait-like qualities, namely the general tendency to become disgusted (9). Both learning processes and disgust sensitivity serve the important function of preventing people from consuming rotten or toxic food (10). Moreover, disgust can be easily generalized, leading associated items of a detestable object to become disgusting themselves (10). Concerning entomophagy, this means that Westeners may have a stereotyped knowledge of insects and other species, and the association of some of those animals with decaying matter and feces could have led to psychological contamination of the entire category (11). However, this does not help to explain why seafood such as crawfish is seen as something “delicious” and regularly enters Westerners' dinner plates whereas insects are seen as something disgusting. Confidence that large-scale behavioral change in favor of insect-based diets is possible may come, for example, from the historical development of the lobster as a luxury product. Once seen as excessive “garbage” in New England (USA), people quip that regulation even existed that lobsters should not be fed to prisoners too frequently. Not only is lobster nowadays a highly rated luxury product, but it is also a key marketing content of New England as a tourist region and hardly an affordable food option for middle-class consumers on a daily base.

Despite the increasing interest in and the great potential of insects as food, scientific knowledge about consumer behavior in the field of entomophagy is largely lacking and has so far primarily focused on correlational studies or hypothetical vignettes [(12), c.f. (13)]. The main aim of the present research is to address this lacuna and to show effective strategies associated with a higher inclination to rely on this source of environmentally friendly and healthy source of animal protein. But how can consumers be approached when one tries to convince them to eat insects? The “as is” strategy of many supporters of entomophagy is to highlight the *environmental* and *health* benefits. This research has placed a high emphasis on the effectiveness of environmental framings on consumer choices, especially in comparison to economic incentives [e.g., (14)]. For instance, this research shows that appealing to environmental motives may outperform appeals to monetary incentives when motivating green behaviors of consumers such as maintaining sufficient tire pressure on one's car. Besides this evidence, a large class of consumer psychological work addresses the effect of environmental or social “labels” (e.g., Fair Trade, eco-friendly) on product judgments [e.g., (15)]. The key result from this research provides robust converging evidence that consumers respond positively to such claims, especially in the domains of “green behaviors” [e.g., (16)]. In addition, research also seems to suggest that “halo effects” of green foods spill over to ratings of healthiness (17, 18). Drawing on this widely known research alone, it is no wonder that many promoters of entomophagy routinely highlight the environmental and health benefits of eating insects, which may seem as the natural candidate for marketing and advertisement campaigns wanting to raise the interest in and willingness to eat insects. And in fact, a plethora of work dealing with entomophagy, not only in the UN FAO report, but also in the popular media ranging from TV documentaries (19) to newspaper articles (20), frequently emphasizes the environmental benefits or the high protein value of insect-based diets. Although these claims are correct and such rational persuading strategies have led to an increase in the awareness of entomophagy benefits, they have barely heightened Westeners' willingness to consume insects (21).

A first skeptical view on the efficacy of Utilitarian claims stems from basic research that links disgust to *executive functions* (22, 23). Executive functions are a set of cognitive processes that are necessary for the cognitive control of behavior. Recent research in environmental behavior calls for a change of the overarching analytical framework to include the role of cognitive control into environmental research and campaigns (24). The key argument is that environmental behavior requires cognitive control by design. Typical environmental-friendly behavior requires foregoing immediate and salient pleasures (e.g., flying to a tropical island, using a car instead of a bike when commuting to work in the rain, not eating excessive amounts of meat, etc.), while the benefits (less CO_2_ emission, more sustainability of resources, etc.) are temporally distant. Also, insect consumption is currently framed as an experience with hardly any immediate rewards, but instead, with long-term utility such as being healthy or being environmentally friendly. Thus, even if consumers were in principle motivated to eat insects for environmental reasons, this process would require certain levels of cognitive control. However, research has linked disgust to a decrease in inhibitory control (25), which is required to make such long-term, goal-oriented decisions. This research suggests that disgusting distracters consume *more* attentional resources and therefore *impair* subsequent inhibitory control to a greater extent than non-disgusting distractors. This would implicate that a decrease in feelings of disgust by hedonic persuasion strategies may also be crucial in order to help individuals to exert the self-control needed for consuming insects for utilitarian reasons. In other words: If insects are perceived as disgusting, as much research suggests (11), highlighting the long-term benefits may not lead a higher willingness-to-consume.

Another explanation for the lack of success of rational persuasion strategies may lie in the fact that attitudes are not only based on rational thoughts and beliefs but also on emotions and feelings. Several researchers [e.g., (26, 27)] have shown that if an attitude is relatively more cognitive or affective in nature has important implications: Attitudes that are based on emotions can generally be changed more effectively with emotional messages than with more cognitive and rational claims. As discussed earlier, central to the aversion toward insects is the feeling of disgust that it evokes in consumers. From the findings of Fabrigar and Petty (26), it directly follows that people's low willingness to eat insects could be influenced more effectively by emotional or hedonic (i.e., insects are tasty) compared to Utilitarian arguments (i.e., our planet needs to be protected). Moreover, research has demonstrated that inducing positive affect in turn can increase participants' ability to make Utilitarian judgements (28). Applying this argument to the present context, participants may be more likely to respond to Utilitarian claims when positive feelings for entomophagy have already been developed. Research on attitudes and ambivalence provides a possible explanation for this effect: People can experience both positive and negative emotions toward an attitude object, which leads to an inner conflict that is particularly apparent when it comes to attitude-relevant decision-making (29). The advantage of this ambivalence is, that it results in enhanced information processing in order to resolve the inner tension (30), and thereby people are more susceptible to new information. Hence, hedonic advertisement may - due to positive change in attitude and potentially stronger information processing - lead to enhanced willingness to try insects.

**Figure 1:**
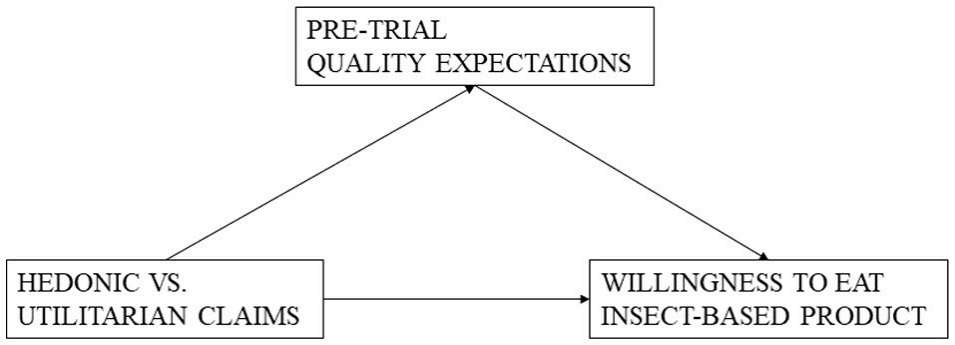
Mediation Model. Link between hedonic persuasion messages and willingness to try inset-based products mediated by enhanced product expectations.

Another crucial channel through which preference development works is pre-trial product expectations. Consumer research has suggested that much of our judgment happens as a “top-down” approach (31). Typically, consumers form expectations about the quality or other intrinsic, but a-priori unknown product characteristics (taste, smell, overall liking, etc.) and, subsequently tend to adhere to their expectation. Because of the strong relationship between pre-trial expectations and product preference, it may prove particularly useful to aim at expectations. As consistency is one of the central drivers of judgment and decision making (32), it is not surprising that many marketing actions (e.g., pricing decisions, packaging, branding, advertisement content) actually aim at raising product expectations, which then easily translate into corresponding preference judgments due to consistency motives by the consumer. Therefore, one suitable channel through which the willingness to eat an insect may be influenced is expectations. Much research supports this process in other food domains. For instance, a study found that knowing before (hence, relevant for expectation building) consumption that a beer is laced with vinegar is detrimental for taste reportings, but learning after tasting does not bias expectations and therefore results in higher taste ratings (33). Furthermore, neuroscientific studies support this reasoning by showing that knowledge about high vs. low prices in wine-tastings leads to brain reactions that already predispose a respective judgment (34). With regard to our study, this means, that the increase in willingness to try insect-based products by hedonic persuasion messages may be mediated by enhanced product expectations (see Figure 1).

To sum up, our central hypothesis is that disgust-based aversions to insects as food are best counterstruck with appeals to hedonic experience rather than by Utilitarian arguments that speak to long-term preferences such as a sustainability of the planet or the healthiness. Moreover, we hypothesize that pre-trial quality expectations mediate this effect. Also, we control for individual differences in disgust sensitivity as well as gender differences, two variables previously associated with insect-consumption (see Figure 1).

## Methods and procedure

### Participants and recruiting procedure

Our experiment was run with a total of 180 volunteer participants (M_age_ = 24.7, SD_age_ = 8.13, 63 percent females) ranging between 18 and 72 years. The large majority (N = 158) was of German nationality and well-educated (115 participants had graduated from high school, 32 from university). The participants had different occupational backgrounds. All were recruited on a centrally located and highly frequented square in Cologne, Germany, to capture a suitable cross-section of the city's population. None of the participants was directly incentivized for eating insect-based foods, but instead, all of them received a flat monetary compensation (€5.00, for about 15–20 min) for their participation in a consumer study. Participants were held unaware during recruiting that the study involved an opportunity to eat an insect. Instead, they were merely recruited for a consumer study focusing on “new products.” As our interest was in the general willingness to consume an insect, this recruiting strategy left our sample unbiased in a sense that people with a particular interest or particularly high levels of disgust could not self-select into or opt-out of the study at this point. Randomization checks in terms of gender, age, previous consumption, and individual difference in disgust sensitivity result in the fact that no important variable was overrepresented in a particular experimental group (gender: *p* = 0.958, age: *p* = 0.616, previous insect consumption: *p* = 0.145, disgust sensitivity: *p* = 0.693).

Participants gave written informed consent and learnt about benefits and risks of the study. The local ethics committee approved the study without a protocol number and further ethical approval was not required. Participants also received the information that people with certain allergic reactions (seafood, gluten, lactose, and nuts/chocolate) as well as pregnant women could not participate in the study due to the novelty of the food and lacking research into potentially adverse effects of insects as food. If a person announced to be allergic or pregnant, s/he received the monetary compensation and was dismissed from the study. People reporting allergic reactions were very rare. Our stopping rule for the sample size was to recruit another participant in case someone reported allergies or pregnancy so that the final samples would be exactly 180 participants (i.e., ~30 per advertisement, therefore, ~60 in the Utilitarian condition and ~120 in the hedonic condition). The study followed all rules of the Declaration of Helsinki. The study was conducted in German.

### Experimental procedure

Upon arrival at the laboratory, participants received oral and written instructions. After consenting, participants worked through a questionnaire that first gave general information about insects as food. The key experimental manipulation was the presentation of an information sheet. In that sheet, participants were confronted with an advertisement flyer of a start-up company planning to enter the entomophagy market. The key sentence on the advertisement was manipulated and always included the statement: “Eating meat has never been so […].” The sentence concluded with one of two types of manipulations, tapping into hedonic reason (dummy coded as 0) vs. Utilitarian reasoning (dummy coded as 1). In the Utilitarian information flyers, the sentence concluded with a random presentation of the words “good for the body” (i.e., healthy), “good for the environment” (i.e., environmentally friendly), or “exquisite” (i.e., highlighting status-oriented consumption). These are the main Utilitarian reasons currently employed by promoters of entomophagy. Tapping into hedonic claims, we used various alternative randomly presented words (delicious, exotic, or trendy).

Participants were asked to engage with the advertisement by writing a short statement about what they saw and what they thought about the informational flyer. The reason for this was that we thus could assure that participants perceive the information and spend some time thinking about the advertisement. After completing this task and some additional questions about their consumption habits (frequency of insect, beef, poultry, and vegetarian consumption), participants received an opportunity to try a mealworm truffle.

Each truffle consisted of a cluster made with ~20 mealworms covered in dark chocolate. The truffles were always presented on a small ceramic plate and participants received a glass on non-carbonated water alongside their food sample. The consumption opportunity was accompanied by a questionnaire in which we first assessed quality expectations by following item: “On the basis of the information available, what quality do you expect from this truffle?”. Participants rated this item on a 7-point scale ranging from 1 = *very bad* to 7 = *very good*. In the second question, participant indicated their willingness to consume the chocolate by either ticking off *yes, I am ready* or *No, I am not ready* and were instructed to follow their choice, serving as our central dependent variable. After voluntary consumption, we assessed participants' general subjective taste ratings (1 = *worst possible truffle* to 11 = *best possible truffle*) and they were asked for a price that they were willing to pay for a 100 g package of the product in the supermarket. Finally, participants completed a 27-item questionnaire assessing disgust sensitivity (35). Thereof, 14 items were rated on a 5-point scale ranging from 0 = *Strongly disagree (very untrue about me)* to 4 = *Strongly agree (very true about me)* and an example item is “If I see someone vomit, it makes me sick to my stomach.”. The remaining 13 items, such as “You see maggots on a piece of meat in an outdoor garbage pail,” were answered on a 5-point scale ranging from 0 = *Not disgusting* at all to 4 = *Extremely disgusting*. Also consumption habits (e.g., “In which supermarket do you prefer to shop?”) as well as demographic variables were assessed. While participants completed the post-experimental questionnaire, the lab assistant prepared the payoff for the participant, paid him or her and dismissed the participant from the study, while also ensuring that the eating decision has been properly noticed (e.g., no difference occurred between an intention to eat the truffle and actual consumption behavior).

## Results

### Preliminary analysis note

Because our research assistants directly approached prospective participants on campus, we sometimes recruited small groups of people. To avoid any effects of social influence, each participant sat at an individual table. Nevertheless, we analyse all our data with clustered standard errors at the session level to control for potential non-independence of observations. Not using clustered standard errors shows largely identical effects and does not alter the interpretation of the results. All analyses were performed using the software SPSS.

### Effects on willingness-to-eat

**Figure 2:**
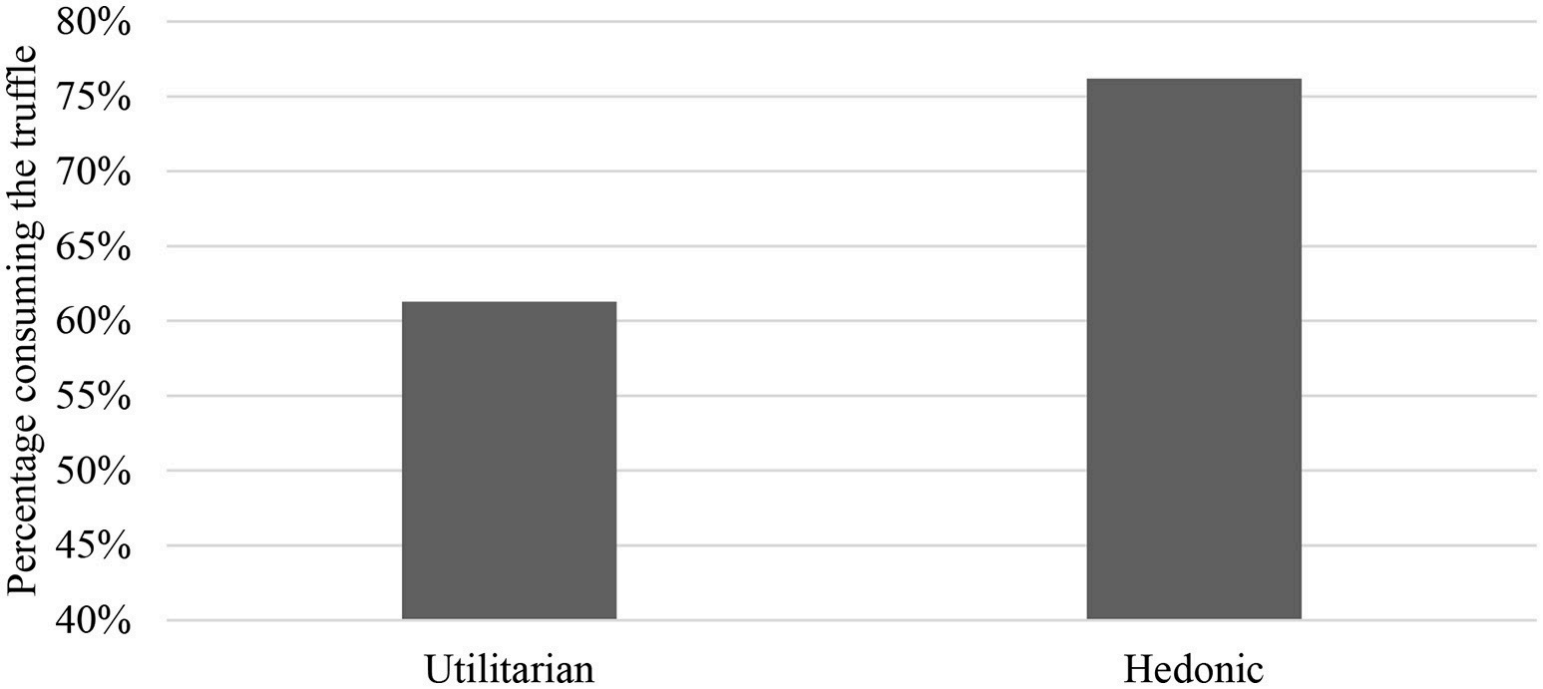
Main results. Percentages of consuming the mealworm truffle (hedonic vs. Utilitarian claims).

First, we analyse the conditional probability of participants' willingness to consume the mealworm-truffle by Utilitarian vs. hedonic advertisement. The difference in the willingness-to-eat is statistically significant (*P* = 0.035, obtained from probit regression using clustered standard errors at the session level) and relevant in terms of effect size (76.2% for hedonic reasons; 61.3% for health and environmental claims combined, health claims alone: 56.6%, environmental claims alone: 65.6%). Figure 2 depicts the result.

**Figure 3:**
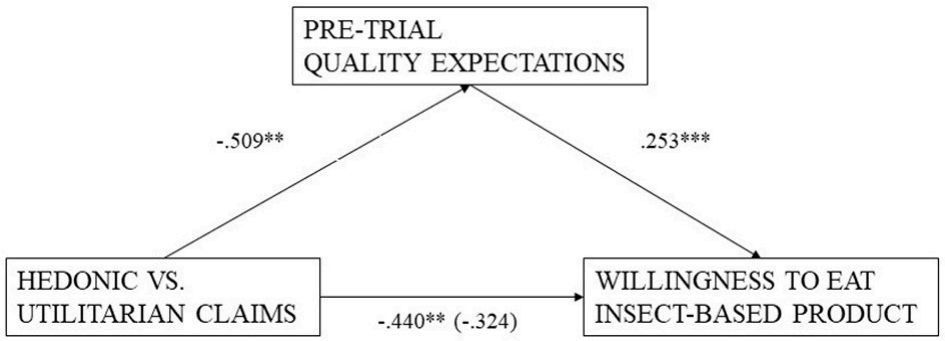
Mediation results. Unstandardized regression coefficients for the relationship between advertising content and willingness to eat an insect-base product as mediated by pre-trial quality expectations. The coefficient between advertising content and willingness to eat insect-based product, controlling for pre-trial quality expectations, is in parantheses, ^***^p < 0.01, ^**^p < 0.05.

Turning to process-evidence, we evaluated how quality expectations mediate the effect of Utilitarian claims on actual consumption using a series of probit and ordinary least squares (OLS) regressions and the bootstrapping procedure recommended by Preacher and Hayes (36). Figure 3 displays all regression results: First, there is a significant negative relationship between Utilitarian claims on willingness to eat (*p* < 0.05). Second, expectations significantly and positively predict the willingness-to-eat (*p* < 0.001, obtained from probit regression using clustered standard errors at the session level). Third, Utilitarian claims significantly negatively impact product expectations (*p* = 0.013, obtained from linear regression using clustered standard errors at the session level). Finally, in a model that includes both, the experimental condition as well as the expectations, the Utilitarian claims no longer predict the willingness-to-eat (*p* = 0.14), but the expectations do so highly significantly (*p* < 0.001, all values obtained from probit regression using clustered standard errors at the session level). To test for this indirect effect directly, we then employed a bootstrapping method. However, to the best of our knowledge, this software does not allow calculation of clustered standard errors. Nevertheless, this mediation using 5,000 re-samples shows a significant indirect effect of Utilitarian claims on the willingness-to-eat via pre-trial quality expectations (effect: −0.2343, boot SE = 0.1198, 95% accelerated and corrected confidence interval = [−0.53; −0.06]).

As an initial summary, the results support the hypothesis that Utilitarian campaigns are less effective to their hedonic counterparts and that pre-trial expectations mediate this relationship. The results, therefore, are consistent with the moral and cognitive psychological research and refute the state-of-the-art approach in current insect marketing. As was shown for many products before, expectations provide a crucial mediator explaining why information content becomes behaviorally relevant.

### Additional analyses: effects on subjective taste ratings and self-reported willingness-to-pay

Next, we analyzed whether subjective taste ratings were also affected by the differentiated campaign focusing on either Utilitarian claims or hedonic claims. Because taste ratings can only be administered when a participant decided to actually try the truffle, this analysis is based on a reduced sample of those actually consuming the product. We observed a marginally significant effect indicating that Utilitarian claims are negatively related to participants subjective taste ratings (coefficient: −0.7428, *P* = 0.07, obtained from linear regression using clustered standard errors at the session level). As was the case for the willingness-to-eat, this effect rendered insignificant once including pre-trial expectations as a mediator. A direct assessment of the indirect effect using bootstrapping methodology corroborates this finding at the marginal significance level (effect: −0.1959, boot SE = 0.1622, 90% accelerated and corrected confidence interval = [−0.56; −0.02]). Turning to self-reported, not incentivized assessments of willingness-to-pay, we do not identify any significant effects of the experimental manipulation.

## General discussion

The present research addresses the efficacy of the state-of-the-art approach to insect marketing, namely to highlight associate environmental or health benefits. The central result is that a shift to “hedonic” campaigns may be better suited to boost insect consumption. Participants were more likely to consume a mealworm truffle when this was advertised in a hedonic way. This finding is in line with results in the promotion of vegetables (37) where hedonic claims outperformed health-related information. Importantly and consistently with other research on consumer products [e.g., (33, 34)], we found that this effect is mediated by pre-trial expectations created when consumers initially engage with the advertisement. The same tendency was also found for taste effects. When insect truffles were marketed as “hedonic,” experimental participants tended to like them better, following higher expectations.

These results challenge the effectiveness of existing campaigns that aim to promote insect consumption by highlighting its environmental and health benefits. Rather, our findings suggest that interventions emphasizing the delicious and unique culinary experience lead to a higher increase in insect consumption. However, further research is needed to confirm whether hedonic interventions are equally effective in non-laboratory settings (e.g., in restaurants, grocery stores, on the product packaging). It is plausible that potential consumers are generally reluctant to buy insect products on a regular basis due to their high price, which is comparable to beef. The reason for this lies in the high need for manual labor for producing edible insect protein up to now, but it is possible that increased demand will drive the development of rearing, harvest, and post-harvesting processing technologies, which in turn will reduce production costs.

Naturally, our results are limited due to several factors. First, we relied on a single market. Although we deliberately opted for a broad sample rather than a typical sample of undergraduate students, our results are essentially mute on the validity in other, unrelated markets. Importantly, effects may not necessarily be transferable to other cultures, countries, or even regions within one country. Furthermore, another limitation comes from the use of merely one product. Although this criticism applies to most of consumer research, it is important to state. However, in related research in which we use several products (13), we find high correlations of eating behavior across products. Put differently, it seems critical to motivate insect consumption in general, but once a person is willing to eat mealworm truffles, s/he is also prone to try mealworm burgers or other products.

Second, our research relied on advertisement campaigns that give very limited information. It could well be that deep information campaigns that—for example—transmit detailed environmental or health information, are better suited to convince prospective consumers about the attractiveness of insects as food. However, this seems rather unrealistic in terms of actual marketing campaigns. Typically, consumers attention (e.g., in stores, in TV, online) is rather limited and it is unlikely that they will engage in intensive information searches. Rather, insect companies will most likely use quick information campaigns (e.g., labels, coloring of packaging) to signal the healthiness or environmental friendliness of their product. In this sense, the current example was highly accurate.

As a summary, our results show that hedonic claims of insect-based products lead to higher expectations, which then result in higher consumption probability and higher taste ratings. Based on these findings, we propose using hedonic instead of utilitarian messages when advertising insect-based foods.

## Ethics statement

This study was carried out in accordance with the recommendations of the local committee at Fresenius University of Applied Science (Cologne Campus) and did not require additional approval. All subjects gave written informed consent in accordance with the Declaration of Helsinki.

## Author contributions

SB, CB, and FC designed the research. CB and CS performed the research. SB, CB, FC, and AW analyzed the data. SB and AW drafted the manuscript. CB, FC, and CS gave feedback.

### Conflict of interest statement

This research originated as the master thesis of CB. Since then, CB has co-founded ESSENTO FOOD AG, a start-up focusing on entomophagy in Switzerland. SB, CS, FC, and AW are not nor intend to be tied in any way to ESSENTO FOOD AG. The reviewer EL and handling editor declare their shared affiliation.
